# Association Between Angiotensin-Converting Enzyme Inhibitors, Angiotensin Receptor Blockers, and Suicide

**DOI:** 10.1001/jamanetworkopen.2019.13304

**Published:** 2019-10-16

**Authors:** Muhammad Mamdani, Tara Gomes, Simon Greaves, Selina Manji, David N. Juurlink, Mina Tadrous, Sidney H. Kennedy, Tony Antoniou

**Affiliations:** 1 Li Ka Shing Knowledge Institute, St Michael’s Hospital, Toronto, Ontario, Canada; 2 Faculty of Medicine, University of Toronto, Toronto, Ontario, Canada; 3 Leslie Dan Faculty of Pharmacy, University of Toronto, Toronto, Ontario, Canada; 4 Institute of Health Policy, Management, and Evaluation, University of Toronto, Toronto, Ontario, Canada; 5 ICES, Toronto, Ontario, Canada; 6 King Saud University, Riyadh, Saudi Arabia; 7 School of Pharmacy, University of Waterloo, Waterloo, Ontario, Canada; 8 Department of Medicine, Sunnybrook Health Sciences Centre, Toronto, Ontario, Canada; 9 Sunnybrook Research Institute, Toronto, Ontario, Canada; 10 Department of Psychiatry, University Health Network, Toronto, Ontario, Canada; 11 Department of Psychiatry, St Michael’s Hospital, Toronto, Ontario, Canada; 12 Krembil Research Institute, University Health Network, Toronto, Ontario, Canada; 13 Department for Family and Community Medicine, University of Toronto, Toronto, Ontario, Canada

## Abstract

**Importance:**

The renin-angiotensin system has been implicated in mood disorders. Angiotensin-converting enzyme inhibitors (ACEIs) and angiotensin receptor blockers (ARBs) are among the most commonly used medications, yet their effects on mental health outcomes, particularly suicide, are poorly understood. This study examined the association between suicide and exposure to ACEIs and ARBs. Because of differences in their mode of action, it was speculated that ARBs would be associated with a higher risk of suicide than ACEIs.

**Objective:**

To examine the association between suicide and exposure to ARBs compared with ACEIs.

**Design, Setting, and Participants:**

This population-based nested case-control study of individuals aged 66 years and older used administrative claims databases in Ontario, Canada, from January 1, 1995, to December 31, 2015. Data analysis was performed from January to April 2019. Cases were individuals who died by suicide within 100 days of receiving an ACEI or ARB. The date of death served as the index date. For each case, 4 controls were identified and matched by age (within 1 year), sex, and presence of hypertension and diabetes. All individuals received an ACEI or ARB within 100 days before the index date.

**Exposures:**

Use of an ACEI or ARB.

**Main Outcomes and Measures:**

Conditional logistic regression was used to estimate odds ratios for the association between suicide and exposure to ARBs compared with ACEIs.

**Results:**

Nine hundred sixty-four cases were matched to 3856 controls. The median (interquartile range) age of cases and controls was 76 (70-82) years. Most cases (768 [79.7%]) and controls (3068 [79.6%]) were men. Among cases, 260 (26.0%) were exposed to ARBs, and 704 (18.4%) were exposed to ACEIs. Among controls, 741 (74.0%) were exposed to ARBs, and 3115 (81.6%) were exposed to ACEIs. Compared with ACEI exposure, ARB exposure was associated with higher risk of death by suicide (adjusted odds ratio, 1.63; 95% CI, 1.33-2.00). The findings were consistent in a sensitivity analysis excluding individuals with a history of self-harm (odds ratio, 1.60; 95% CI, 1.29-1.98).

**Conclusions and Relevance:**

The use of ARBs may be associated with an increased risk of suicide compared with ACEIs. Preferential use of ACEIs over ARBs should be considered whenever possible, particularly in patients with severe mental health illness.

## Key Points

**Question:**

Are angiotensin receptor blockers associated with a higher risk of suicide than angiotensin-converting enzyme inhibitors?

**Findings:**

In this case-control study of 964 older adults who died by suicide and 3856 matched control participants, compared with angiotensin-converting enzyme inhibitors, exposure to angiotensin receptor blockers was associated with a higher risk of suicide. Findings were consistent in a sensitivity analysis that excluded individuals with a history of self-harm.

**Meaning:**

Angiotensin receptor blockers may be associated with a higher risk of suicide than angiotensin-converting enzyme inhibitors.

## Teaser

This population-based case-control study of individuals aged 66 years and older in Ontario, Canada, examines whether angiotensin receptor blockers are associated with a higher risk of suicide than angiotensin-converting enzyme inhibitors.

## Introduction

Angiotensin-converting enzyme inhibitors (ACEIs) and angiotensin receptor blockers (ARBs) are widely used for the management of hypertension, chronic kidney disease, heart failure, and diabetes. These drugs lower blood pressure by modulating the renin-angiotensin aldosterone system in distinct ways. Angiotensin-converting enzyme inhibitors inhibit the conversion of angiotensin I to angiotensin II (AII), whereas ARBs block the binding of AII to its AII type 1 receptor, resulting in upregulation of AII and unopposed stimulation of the AII type 2 receptor.^1^

Although peripheral AII does not cross the blood-brain barrier, AII is also generated in the central nervous system.^2^ Its central effects include modulation of neurotransmitter release and activation of proinflammatory pathways that may influence mental health.^2,3^ Because ACEI and ARBs can cross the blood-brain barrier to various degrees, these drugs may interfere with central AII activity. The effect of these drugs on mental health outcomes, particularly suicide, is of increasing interest because of the bidirectional association between depression and cardiovascular disease.^4^ Although both drug classes could have anti-inflammatory or neuroprotective effects as an extension of their pharmacological effects, ARB-mediated compensatory increases in brain AII could inadvertently worsen outcomes. This assertion is supported by an increased risk of suicide in patients with *ACE* gene polymorphisms associated with higher levels of this peptide.^5,6^ The mechanisms by which AII may be associated with a higher risk of suicide remain largely unclear. Possible explanations include AII-mediated increases in substance P activity and heightened hypothalamic-pituitary-adrenal axis activity, provoking stress and anxiety.^7,8,9^ Moreover, polymorphisms associated with higher levels of AII have been associated with other mental health conditions, including major depression, bipolar disorder, panic disorder, and anxiety disorder.^7,8,10,11,12^ Furthermore, recent data^13^ suggest that users of ARBs, but not ACEIs, may have an increased risk of suicide compared with nonusers. The objective of our study was to examine the association between suicide and exposure to ARBs compared with ACEIs. We hypothesized that exposure to ARBs would be associated with a higher risk of suicide compared with ACEIs.

## Methods

We conducted a nested case-control study among residents of Ontario, Canada, aged 66 years and older from January 1, 1995, to December 31, 2015. The primary objective was to examine the association between suicide and exposure to ARBs compared with ACEIs. Ontario is Canada’s largest province, with more than 2.3 million elderly residents who have access to publicly funded health care, including physician services, hospital care, and prescription drugs. We used population-based databases documenting health service utilization and outcomes, including the Ontario Drug Benefit database, which captures prescription drugs dispensed; the Canadian Institute for Health Information Discharge Abstract Database, which captures inpatient hospitalization data; the Ontario Health Insurance Plan database, which captures data on physician visits; the Registered Persons Database, which captures basic demographic information; the Ontario Hypertension Database and the Ontario Diabetes Database, which capture data on the prevalence of hypertension and diabetes, respectively; and the Office of the Registrar General–Deaths, which captures information on deaths, including suicides. These databases are anonymously linked using encrypted identifiers and are routinely used for research purposes.^14,15^

The study was approved by the research ethics board of Sunnybrook Health Sciences Centre. ICES is a prescribed entity under section 45 of Ontario’s Personal Health Information Protection Act. Section 45 authorizes ICES to collect personal health information, without consent, for the purpose of analysis or compiling statistical information with respect to the management, evaluation, or monitoring of the allocation of resources to or planning for all or part of the health system. This study follows the Strengthening the Reporting of Observational Studies in Epidemiology (STROBE) reporting guideline for case-control studies.

We defined cases as patients who died by suicide within 100 days of receiving a prescription for an ACEI or ARB, excluding individuals who received drugs from both of these classes during the same period (see the eTable in the Supplement for codes). Both ACEIs and ARBs are publicly funded for older adults in Ontario without limitations or restrictions through Ontario Drug Benefit, which will cover a maximum of 100 days’ supply of each prescription. The date of suicide served as the index date. For each case, we selected up to 4 controls, matching by age (within 1 year of the index date), sex, and previous diagnoses of hypertension and diabetes, and assigned them the same index date as their matched case. We allowed controls to serve as a control more than once or to become a case subsequently. Controls were also required to have been exposed to an ACEI or ARB within 100 days preceding the index date.

### Statistical Analysis

We compared baseline characteristics of cases and controls using standardized differences (ie, the difference in the mean of a variable between 2 groups divided by an estimate of the SD of that variable), where values greater than 0.10 suggest a significant difference between cases and controls,^16^ and used multivariable conditional logistic regression to generate odds ratios and 95% CIs for the association between suicide and exposure to ARBs compared with ACEIs. We adjusted for potential confounders, such as Charlson Comorbidity Index score, socioeconomic status, residence in a long-term care facility, congestive heart failure, and a history of the following in the past year: stroke, chronic kidney disease, chronic liver disease, alcohol abuse, affective disorder, anxiety or sleep disorder, psychoses, agitation and related disorders, other mental health conditions, and specific drug classes as individual terms, including psychotropic drugs, cholesterol-lowering drugs, and other antihypertensives. Because there was a very small number of controls with a history of self-harm (see eTable in the Supplement for codes), we did not adjust for this variable in our primary model. In a sensitivity analysis, we removed all cases and their respective controls with past incidents of deliberate self-harm to test the robustness of the findings. All analyses were conducted using SAS statistical software version 9.2 (SAS Institute) between January and April 2019.

## Results

Over the 18-year study period, we matched 964 cases to 3856 controls exposed to either an ACEI or ARB within 100 days before the index date. Most cases (768 [79.7%]) and controls (3068 [79.6%]) were men, and the median (interquartile range) age was 76 (70-82) years. As expected, comorbidities and psychotropic drug use were more common among cases. For example, cases were more likely than controls to have histories of alcohol abuse (3.3% vs 0.6%; standardized difference, 0.20); anxiety or sleep disorders (42.8% vs 14.7%; standardized difference, 0.65); psychoses, agitation, and related disorders (41.6% vs 14.5%; standardized difference, 0.63); affective disorder (42.6% vs 14.7%; standardized difference, 0.65); and other mental health conditions (42.9% vs 17.7%; standardized difference, 0.66) (Table 1). Cases were also more likely than controls to use antidepressants (38.0% vs 13.3%; standardized difference, 0.59), antipsychotics (11.7% vs 31.%; standardized difference, 0.34), benzodiazepines (40.1% vs 14.0%; standardized difference, 0.62), and mood stabilizers (3.4% vs 1.6%; standardized difference, 0.12). The most common ACEIs were ramipril (38.8%) and enalapril (15.0%). The most common ARBs were valsartan (16.7%), telmisartan (16.7%), and candesartan (16.7%).

**Table 1.** Characteristics of Cases and Matched Controls

Characteristics	Participants, No. (%)		Standardized Difference^a^
	Cases (n = 964)	Controls (n = 3856)	
Age, median (IQR), y	76 (70-82)	76 (70-82)	0.00
Age range, y			
66-74	429 (44.5)	1728 (44.8)	0.01
75-84	396 (41.1)	1587 (41.2)	0.00
≥85	139 (14.4)	541 (14.0)	0.01
Female	196 (20.3)	788 (20.4)	0.00
Urban residence	802 (83.2)	3266 (84.7)	0.04
Charlson Comorbidity Index score^b^			
No hospitalizations	461 (47.8)	2486 (64.5)	0.34
0	158 (16.4)	448 (11.6)	0.14
1	102 (10.6)	355 (9.2)	0.05
≥2	243 (25.2)	567 (14.7)	0.27
Income quintile			
1 (lowest)	213 (22.1)	796 (20.6)	0.04
2	184 (19.1)	803 (20.8)	0.04
3	205 (21.3)	745 (19.3)	0.05
4	170 (17.6)	750 (19.5)	0.05
5	188 (19.5)	745 (19.3)	0.01
Missing	≤5 (0.4)	17 (0.4)	0.00
Residence in long-term care facility	6 (0.6)	141 (3.7)	0.21
Stroke	33 (3.4)	65 (1.7)	0.11
Coronary artery disease	285 (29.6)	1066 (27.6)	0.04
Chronic kidney disease	44 (4.6)	47 (1.2)	0.20
Chronic liver disease	16 (2.0)	27 (0.8)	0.10
Congestive heart failure	245 (25.4)	930 (24.1)	0.03
Hypertension	840 (87.1)	3360 (87.1)	0.00
Diabetes	360 (37.3)	1440 (37.3)	0.00
Alcohol abuse	32 (3.3)	23 (0.6)	0.20
Anxiety or sleep disorders	413 (42.8)	568 (14.7)	0.65
Psychoses, agitation, and related disorders	401 (41.6)	561 (14.5)	0.63
Affective disorder	411 (42.6)	568 (14.7)	0.65
All other mental health conditions	414 (42.9)	568 (14.7)	0.66
Deliberate self-harm	98 (10.2)	0	0.48
Visits, median (IQR), No.			
To psychiatrist	0 (0-0)	0 (0-0)	0.30
To cardiologist	0 (0-0)	0 (0-0)	0.02
Drug use in past year			
Prescription drugs, median (IQR), No.	4 (3-6)	3 (2-4)	0.57
Antidepressants	366 (38.0)	511 (13.3)	0.59
Antipsychotics	113 (11.7)	118 (3.1)	0.34
Benzodiazepines	387 (40.1)	541 (14.0)	0.62
Mood stabilizers^c^	33 (3.4)	61 (1.6)	0.12
Cholesterol-lowering medications^d^	481 (49.9)	2238 (58.0)	0.16
β-adrenergic antagonists	257 (26.7)	1303 (33.8)	0.16
Calcium channel blockers	338 (35.1)	1245 (32.3)	0.06
Other antihypertensives	407 (42.2)	1559 (40.4)	0.04
Potassium-sparing diuretics	67 (7.0)	187 (4.8)	0.09
Loop diuretics	156 (16.2)	587 (15.2)	0.03
Thiazide diuretics	190 (19.7)	796 (20.6)	0.02
Other^e^	59 (6.1)	172 (4.5)	0.07

Abbreviation: IQR, interquartile range.

^a^ Difference in the mean of a variable between 2 groups divided by an estimate of the SD of that variable.

^b^ In the year preceding the index date.

^c^ Includes lithium, divalproex, valproic acid, oxcarbazepine, and carbamazepine.

^d^ Includes statins, fibrates, and resins.

^e^ Includes α-blockers.

Among cases, 260 (26.0%) were exposed to ARBs, and 704 (18.4%) were exposed to ACEIs. Among controls, 741 (74.0%) were exposed to ARBs, and 3115 (81.6%) were exposed to ACEIs. In the primary analysis, compared with ACEIs, ARB exposure was associated with a higher risk of suicide (adjusted odds ratio, 1.63; 95% CI, 1.33-2.00) (Table 2). We observed consistent findings in our sensitivity analysis excluding individuals with a history of deliberate self-harm (odds ratio, 1.60; 95% CI, 1.29-1.98).

**Table 2.** Association Between Angiotensin-Converting Enzyme Inhibitors, Angiotensin Receptor Blockers, and Suicide

Exposure	Patients Exposed, No. (%)		Odds Ratio (95% CI)	
	Cases (n = 964)	Controls (n = 3856)	Unadjusted	Adjusted^a^
Angiotensin receptor blockers	260 (26.0)	741 (74.0)	1.64 (1.37-1.95)	1.63 (1.33-2.00)
Angiotensin-converting enzyme inhibitors	704 (18.4)	3115 (81.6)	1 [Reference]	1 [Reference]

^a^ Adjusted for Charlson Comorbidity Index score, income quintile, residence in a long-term care facility, drug use in preceding year (antidepressants, antipsychotics, benzodiazepines, mood stabilizers, cholesterol-lowering medications, β-adrenergic antagonists, calcium channel blockers, potassium-sparing diuretics, loop diuretics, thiazide diuretics, and other antihypertensives), psychiatrist visits in preceding year, cardiologist visits in preceding year, history of alcohol abuse, stroke, chronic kidney disease in preceding year, chronic liver disease in previous year, congestive heart failure, coronary artery disease in previous year, and other mental health conditions (see Table 1).

## Discussion

We found that ARBs were associated with a higher risk of suicide compared with ACEIs among individuals aged 66 years and older in Ontario, Canada. Previous studies^5,6^ have linked ACE polymorphisms to suicide, and 1 study^13^ reported a greater than 3-fold increased risk of suicide among ARB users compared with nonusers. However, that study was not intended to specifically examine the association between ACEIs, ARBs, and suicide and was limited by a very small number of patients being exposed to ARBs. The mechanisms by which ARBs or ACEIs might impart differential risks of suicide are unknown. As noted earlier, higher levels of AII may increase levels of substance P, which may, in turn, promote stress and anxiety.^7,9^ Similarly, animal studies^17,18,19^ have found that AII induces panic and stress when injected into stress-sensitive brain structures such as the dorsomedial hypothalamus and amygdala. However, because these effects were reversible after AII type 1 receptor blockade with an ARB, it is unlikely that this is the mechanism associating these drugs with suicide. Another possible explanation for a higher risk of suicide among users of ARBs is associated with the upregulation of AII and resulting unopposed stimulation of AII type 2 receptors.^1^ These effects have been associated with nuclear factor–κB pathway activation,^20^ a process increasingly recognized as being involved in the pathophysiology of mood disorders.^21,22^ The mechanisms linking ARBs to mental health conditions and whether these effects are common to all members of this class are areas where further research is required.

### Limitations

The findings of our study should be considered as preliminary and need to be confirmed in future studies. Several limitations to our study should be noted. First, we were unable to assess clinically important comorbidities and behaviors such as substance use, although we do not anticipate any major differences in such characteristics between exposure groups. In addition, we did not have reliable data on mental health–related hospital admissions and emergency department visits. Second, we studied an elderly population, and the generalizability of our findings to younger populations is not known. Similarly, because most cases were men, these findings may not be generalizable to women. Third, suicide may be misclassified, particularly among elderly individuals, although there is no apparent reason why this would be differentially influenced by ACEI or ARB exposure. Furthermore, this would likely bias our findings toward a null hypothesis, and systematic misclassification based on exposure status is unlikely. Fourth, our study had insufficient power to explore potential intraclass differences in suicide risk.

## Conclusions

Our findings suggest a possible increased risk of suicide associated with the use of ARBs compared with ACEIs among adults aged 66 years and older. Given their high prevalence of use, the severity of the outcome, and the similar efficacy of these drug classes in treating the same conditions, clinicians may opt for preferential use of ACEIs over ARBs where possible.

## Notes

Supplement. **eTable.** Codes for Suicide and Self-harm Ascertainment

## References

[1] Strauss MH, Hall AS The divergent cardiovascular effects of angiotensin converting enzyme inhibitors and angiotensin receptor blockers on myocardial infarction and death. Prog Cardiovasc Dis. 2016;58(5):-. doi:10.1016/j.pcad.2015.11.004 26586276

[2] Xu Q, Jensen DD, Peng H, Feng Y The critical role of the central nervous system (pro)renin receptor in regulating systemic blood pressure. Pharmacol Ther. 2016;164:126-134. doi:10.1016/j.pharmthera.2016.04.006 27113409 PMC4942374

[3] Tsuda K Renin-angiotensin system and sympathetic neurotransmitter release in the central nervous system of hypertension. Int J Hypertens. 2012;2012:474870. doi:10.1155/2012/474870 23227311 PMC3512297

[4] Lippi G, Montagnana M, Favaloro EJ, Franchini M Mental depression and cardiovascular disease: a multifaceted, bidirectional association. Semin Thromb Hemost. 2009;35(3):325-336. doi:10.1055/s-0029-1222611 19452408

[5] Hishimoto A, Shirakawa O, Nishiguchi N, Association between a functional polymorphism in the renin-angiotensin system and completed suicide. J Neural Transm (Vienna). 2006;113(12):1915-1920. doi:10.1007/s00702-006-0483-9 16736244

[6] Sparks DL, Hunsaker JC III, Amouyel P, Angiotensin I-converting enzyme I/D polymorphism and suicidal behaviors. Am J Med Genet B Neuropsychiatr Genet. 2009;150B(2):290-294. doi:10.1002/ajmg.b.30793 18521860

[7] Arinami T, Li L, Mitsushio H, Itokawa M, Hamaguchi H, Toru M An insertion/deletion polymorphism in the angiotensin converting enzyme gene is associated with both brain substance P contents and affective disorders. Biol Psychiatry. 1996;40(11):1122-1127. doi:10.1016/S0006-3223(95)00597-8 8931914

[8] Baghai TC, Binder EB, Schule C, Polymorphisms in the angiotensin-converting enzyme gene are associated with unipolar depression, ACE activity and hypercortisolism. Mol Psychiatry. 2006;11(11):1003-1015. doi:10.1038/sj.mp.4001884 16924268

[9] Ebner K, Singewald N The role of substance P in stress and anxiety responses. Amino Acids. 2006;31(3):251-272. doi:10.1007/s00726-006-0335-9 16820980

[10] Kucukali CI, Aydin M, Ozkok E, Angiotensin-converting enzyme polymorphism in schizophrenia, bipolar disorders, and their first-degree relatives. Psychiatr Genet. 2010;20(1):14-19. doi:10.1097/YPG.0b013e3283351194 20010451

[11] Erhardt A, Lucae S, Kern N, Association of polymorphisms in the angiotensin-converting enzyme gene with syndromal panic attacks. Mol Psychiatry. 2008;13(3):242-243. doi:10.1038/sj.mp.4002094 18285758

[12] Saab YB, Gard PR, Yeoman MS, Mfarrej B, El-Moalem H, Ingram MJ Renin-angiotensin-system gene polymorphisms and depression. Prog Neuropsychopharmacol Biol Psychiatry. 2007;31(5):1113-1118. doi:10.1016/j.pnpbp.2007.04.002 17499413

[13] Callréus T, Agerskov Andersen U, Hallas J, Andersen M Cardiovascular drugs and the risk of suicide: a nested case-control study. Eur J Clin Pharmacol. 2007;63(6):591-596. doi:10.1007/s00228-007-0293-5 17468865

[14] Finkelstein Y, Macdonald EM, Hollands S, ; Canadian Drug Safety and Effectiveness Research Network (CDSERN) Risk of suicide following deliberate self-poisoning. JAMA Psychiatry. 2015;72(6):570-575. doi:10.1001/jamapsychiatry.2014.3188 25830811

[15] Zipursky JS, Macdonald EM, Luo J, ; Canadian Drug Safety and Effectiveness Research Network Lipophilic β-blockers and suicide in the elderly. J Clin Psychopharmacol. 2017;37(3):381-384. doi:10.1097/JCP.0000000000000695 28338548

[16] Mamdani M, Sykora K, Li P, Reader’s guide to critical appraisal of cohort studies: assessing potential for confounding. BMJ. 2005;330(7497):960-962. doi:10.1136/bmj.330.7497.960 15845982 PMC556348

[17] Shekhar A, Johnson PL, Sajdyk TJ, Angiotensin-II is a putative neurotransmitter in lactate-induced panic-like responses in rats with disruption of GABAergic inhibition in the dorsomedial hypothalamus. J Neurosci. 2006;26(36):9205-9215. doi:10.1523/JNEUROSCI.2491-06.2006 16957077 PMC6674511

[18] Shekhar A, Sajdyk TJ, Gehlert DR, Rainnie DG The amygdala, panic disorder, and cardiovascular responses. Ann N Y Acad Sci. 2003;985(Apr):308-325. doi:10.1111/j.1749-6632.2003.tb07090.x 12724167

[19] Liu F, Havens J, Yu Q, The link between angiotensin II-mediated anxiety and mood disorders with NADPH oxidase-induced oxidative stress. Int J Physiol Pathophysiol Pharmacol. 2012;4:28-35. 22461954 PMC3312460

[20] Wolf G, Wenzel U, Burns KD, Harris RC, Stahl RAK, Thaiss F Angiotensin II activates nuclear transcription factor-kappaB through AT1 and AT2 receptors. Kidney Int. 2002;61(6):1986-1995. doi:10.1046/j.1523-1755.2002.00365.x 12028439

[21] Coon H, Darlington T, Pimentel R, Genetic risk factors in two Utah pedigrees at high risk for suicide. Transl Psychiatry. 2013;3:e325. doi:10.1038/tp.2013.100 24252905 PMC3849959

[22] Miklowitz DJ, Portnoff LC, Armstrong CC, Inflammatory cytokines and nuclear factor-kappa B activation in adolescents with bipolar and major depressive disorders. Psychiatry Res. 2016;241:315-322. doi:10.1016/j.psychres.2016.04.120 27227701 PMC4912920

