# Errors in Abstract, Results, and Table 2

**DOI:** 10.1001/jamanetworkopen.2020.1382

**Published:** 2020-02-28

In the Original Investigation titled “Association Between Angiotensin-Converting Enzyme Inhibitors, Angiotensin Receptor Blockers, and Suicide,”^1^ published October 16, 2019, there were errors in the percentages of cases and controls exposed to angiotensin-converting enzyme inhibitors and angiotensin receptor blockers shown in the Abstract, Results, and Table 2. Among cases, 27.0% were exposed to angiotensin receptor blockers, and 73.0% were exposed to angiotensin-converting enzyme inhibitors. Among controls, 19.2% were exposed to angiotensin receptor blockers, and 80.8% were exposed to angiotensin-converting enzyme inhibitors. This article has been corrected.^1^
