# Supplementary material for: Association Between Angiotensin-Converting Enzyme Inhibitors, Angiotensin Receptor Blockers, and Suicide
Source: JAMA Netw Open. 2019 Oct 16;2(10):e1913304. doi: 10.1001/jamanetworkopen.2019.13304 (PMC6806420; doi:10.1001/jamanetworkopen.2019.13304)
Supplement: Supplement. — eTable. Codes for Suicide and Self-harm Ascertainment [file jamanetwopen-2-e1913304-s001.pdf]

Supplementary Online Content

Mamdani M, Gomes T, Greaves S, et al. Association between angiotensin-converting enzyme inhibitors, angiotensin receptor blockers, and suicide. *JAMA Netw Open*. 2019;2(10):e1913304. doi:10.1001/jamanetworkopen.2019.13304

eTable. Codes for Suicide and Self-harm Ascertainment

This supplementary material has been provided by the authors to give readers additional information about their work.

© 2019 Mamdani M et al. *JAMA Network Open.*

eTable. Codes for Suicide and Self-harm Ascertainment

Outcome	Codes
Suicide	E950 External Cause - Suicide & Self-Inflicted Injury<br>E951 External Cause - Suicide & Self-Inflicted Injury<br>E952 External Cause - Suicide & Self-Inflicted Injury<br>E953 External Cause - Suicide & Self-Inflicted Injury<br>E954 External Cause - Suicide & Self-Inflicted Injury<br>E955 External Cause - Suicide & Self-Inflicted Injury<br>E956 External Cause - Suicide & Self-Inflicted Injury<br>E957 External Cause - Suicide & Self-Inflicted Injury<br>E958 External Cause - Suicide & Self-Inflicted Injury<br>E959 External Cause - Suicide & Self-Inflicted Injury
Self-harm	X60 Intentional self-poisoning by and exposure to nonopioid analgesics, antipyretics and antirheumatics<br>X61 Intentional self-poisoning by and exposure to antiepileptic, sedative-hypnotic, antiparkinsonism and psychotropic drugs, not elsewhere classified<br>X62 Intentional self-poisoning by and exposure to narcotics and psychodysleptics [hallucinogens], not elsewhere classified<br>X63 Intentional self-poisoning by and exposure to other drugs acting on the autonomic nervous system<br>X64 Intentional self-poisoning by and exposure to other and unspecified drugs, medicaments and biological substances<br>X65 Intentional self-poisoning by and exposure to alcohol<br>X66 Intentional self-poisoning by and exposure to organic solvents and halogenated hydrocarbons and their vapours<br>X67 Intentional self-poisoning by and exposure to other gases and vapours<br>X68 Intentional self-poisoning by and exposure to pesticides<br>X69 Intentional self-poisoning by and exposure to other and unspecified chemicals and noxious substances<br><br>X70 Intentional self-harm by hanging, strangulation and suffocation<br>X71 Intentional self-harm by drowning and submersion<br>X72 Intentional self-harm by handgun discharge<br>X73 Intentional self-harm by rifle, shotgun and larger firearm discharge<br>X7400 Intentional self-harm by BB gun discharge

© 2019 Mamdani M et al. *JAMA Network Open.*

	X7401 Intentional self-harm by air gun discharge<br>X7408 Intentional self-harm by other specified firearm discharge<br>X7409 Intentional self-harm by unspecified firearm discharge<br>X75 Intentional self-harm by explosive material<br>X76 Intentional self-harm by smoke, fire and flames<br>X77 Intentional self-harm by steam, hot vapours and hot objects<br>X78 Intentional self-harm by sharp object<br>X79 Intentional self-harm by blunt object<br>X80 Intentional self-harm by jumping from a high place<br>X81 Intentional self-harm by jumping or lying before moving object<br>X82 Intentional self-harm by crashing of motor vehicle<br>X83 Intentional self-harm by other specified means<br>X84 Intentional self-harm by unspecified means<br>Z915 Personal history of self-harm

© 2019 Mamdani M et al. *JAMA Network Open.*
